# Artificial Intelligence Enabled Personalised Assistive Tools to Enhance Education of Children with Neurodevelopmental Disorders—A Review

**DOI:** 10.3390/ijerph19031192

**Published:** 2022-01-21

**Authors:** Prabal Datta Barua, Jahmunah Vicnesh, Raj Gururajan, Shu Lih Oh, Elizabeth Palmer, Muhammad Mokhzaini Azizan, Nahrizul Adib Kadri, U. Rajendra Acharya

**Affiliations:** 1 School of Business, University of Southern Queensland, Springfield 4300, Australia; Prabal.Barua@usq.edu.au (P.D.B.); Raj.Gururajan@usq.edu.au (R.G.); 2 Faculty of Engineering and Information Technology, University of Technology, Sydney 2007, Australia; 3 Department of Electronics and Computer Engineering, Ngee Ann Polytechnic, Singapore 599489, Singapore; e0145834@u.nus.edu (J.V.); shulih@hotmail.com (S.L.O.); aru@np.edu.sg (U.R.A.); 4 School of Woman’s and Children’s Health, University of New South Wales, Sydney 2031, Australia; elizabeth.palmer@unsw.edu.au; 5 Centre for Clinical Genetics, Sydney Children’s Hospital, Randwick, New South Wales 2031, Australia; 6 Faculty of Engineering and Built Environment, Universiti Sains Islam Malaysia, Bandar Baru Nilai, Nilai 71800, Malaysia; 7 Department of Biomedical Engineering, Faculty of Engineering, University Malaya, Kuala Lumpur 50603, Malaysia; nahrizuladib@um.edu.my; 8 School of Science and Technology, Singapore University of Social Sciences, Singapore 599494, Singapore; 9 Department of Bioinformatics and Medical Engineering, Asia University, Taichung City 41354, Taiwan

**Keywords:** neurodevelopmental disorders, mental disorders, personalisation, artificial intelligence, machine learning

## Abstract

Mental disorders (MDs) with onset in childhood or adolescence include neurodevelopmental disorders (NDDs) (intellectual disability and specific learning disabilities, such as dyslexia, attention deficit disorder (ADHD), and autism spectrum disorders (ASD)), as well as a broad range of mental health disorders (MHDs), including anxiety, depressive, stress-related and psychotic disorders. There is a high co-morbidity of NDDs and MHDs. Globally, there have been dramatic increases in the diagnosis of childhood-onset mental disorders, with a 2- to 3-fold rise in prevalence for several MHDs in the US over the past 20 years. Depending on the type of MD, children often grapple with social and communication deficits and difficulties adapting to changes in their environment, which can impact their ability to learn effectively. To improve outcomes for children, it is important to provide timely and effective interventions. This review summarises the range and effectiveness of AI-assisted tools, developed using machine learning models, which have been applied to address learning challenges in students with a range of NDDs. Our review summarises the evidence that AI tools can be successfully used to improve social interaction and supportive education. Based on the limitations of existing AI tools, we provide recommendations for the development of future AI tools with a focus on providing personalised learning for individuals with NDDs.

## 1. Introduction

### 1.1. Mental Disorders

Mental disorders with onset in childhood or adolescence, as encapsulated by the Diagnostic and Statistical Manual of Mental Disorders DSM-V, include both neurodevelopmental disorders (NDDs), such as intellectual disability; specific learning disabilities, such as dyslexia, attention deficit hyperactivity disorder (ADHD) and autism spectrum disorders (ASDs); and mental health disorders (MHDs), such as depressive, anxiety, stress related, conduct and psychotic disorders. Globally, childhood-onset mental disorders are becoming more prevalent, with a rate of 10–20% [1]. NDDs are now recognised as a leading cause of morbidity in children, with a huge impact not only on the individual child, but also on their families and society [2]. There is also a possibility of the development of suicidal ideation in the aforementioned depressive [3], anxiety [4], stress-related [5], conduct [6] and psychotic disorders [4]. Moreover, there is frequent overlap between NDDs and MHDs. For example, between 30% and 50% of children with ADHD are reported to have a comorbid MHD, such as depression [7,8], and over 70% of children with ASD are reported to have a comorbid MHD, most commonly including anxiety (40%) and ADHD (30–40%) [9,10]. The complex web of comorbidities between NDDs and MHDs can contribute to the severity of learning disabilities [11] in individual children and can further reduce the quality of life. This complex comorbidity can be associated with poorer long-term prognosis and has implications for tailoring treatments for individual children [12].

Both genetics and environmental factors contribute to the development of MDs in children. A child’s neurological functioning and mental health can be influenced by the family’s socioeconomic status, wherein poverty and low family income can affect the education, health and self-esteem of children, adversely affecting their growth, development and societal involvement. Furthermore, a parent’s own poor health, mental disorder, social isolation or housing insecurity can diminish his/her capability of effectively parenting the child by providing a supportive environment for growth and development, which is necessary to keep mental health problems at bay [13].

Thus, childhood onset MDs in general, and NDDs specifically, have a huge impact on individuals, families and society, and there is a pressing need to understand how best to recognise and treat these conditions. This systematic review has two aims. First, to provide an overview of how AI technologies have been applied to assist students with the most common and impactful NDDs, namely ADHD, dyslexia and ASDs. Second, to highlight the limitations of existing developed AI tools to enable recommendations to be made for future directions in AI development, so that personalised education for these students can be improved further.

#### 1.1.1. ADHD

**Figure 1:**
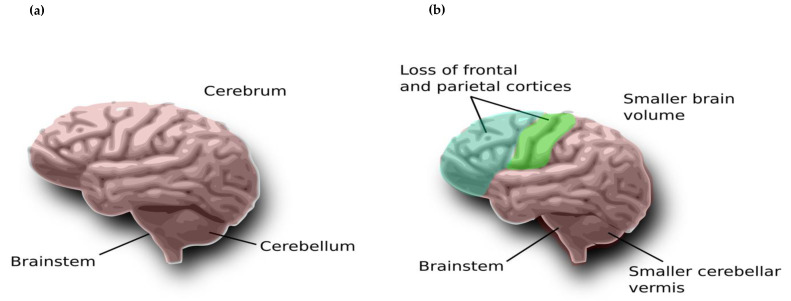
(**a**) Normal brain and (**b**) ADHD brain with smaller volume.

The NDD ADHD, with prevalence reported between 9% and 40% [14], has multiple aetiologies wherein combinations of environmental and genetic aspects play a role in contributing to the pathogenesis and its diverse phenotypes [15]. Non-genetic risk factors have been suggested to include brain injury, premature delivery, maternal alcohol and tobacco use during pregnancy, and contact with certain environmental agents, such as lead, during pregnancy or at a young age [16,17,18]. Not uncommonly, children meeting diagnostic criteria for ADHD have other comorbidities, including oppositional defiant and conduct disorders (ODD and CDs) [19,20] and depressive and anxiety disorders [21], as well as specific learning disorders [22]. Children with ADHD have difficulties in maintaining sustained attention, can be hyperactive, fidget and find it difficult to participate in turn taking [17]. Preliminary research points to differences in brain volumes in children diagnosed with ADHD compared to neurotypical children, particularly affecting the frontal and parietal cortices (Figure 1).

#### 1.1.2. Dyslexia

**Figure 2:**
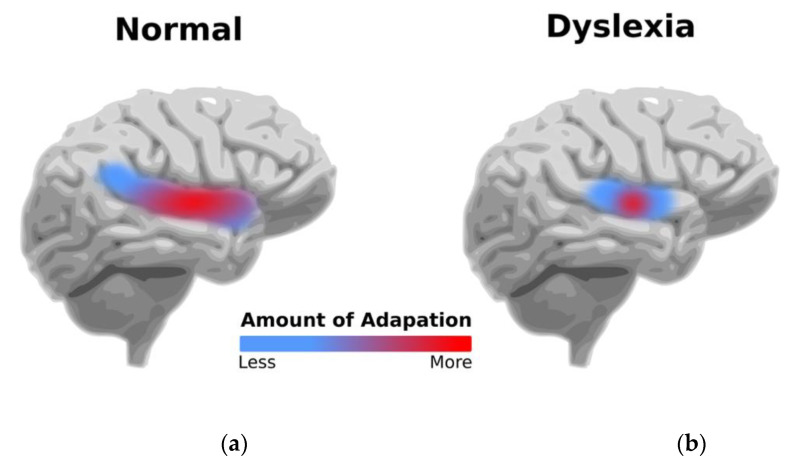
(**a**) Large neural adaptation in normal brain and (**b**) reduced neural adaptation in dyslexic brain.

NDD dyslexia is a common type of learning disability, affecting 3 to 15% of school-age children [23]. Individuals with dyslexia have specific impairments in the development of expert reading skills. Dyslexia is characterised by difficulties with correct and/or fluent word recognition and poor spelling and decoding abilities [24]. Individuals with dyslexia have been shown to have differences in functional brain imaging compared to non-dyslexic individuals, for example, reduced neural adaptation to repetitive stimuli [25], as depicted in Figure 2. Children with dyslexia can have other specific learning deficits [24], low self-confidence, anxiety and depression.

#### 1.1.3. Autism Spectrum Disorders

**Figure 3:**
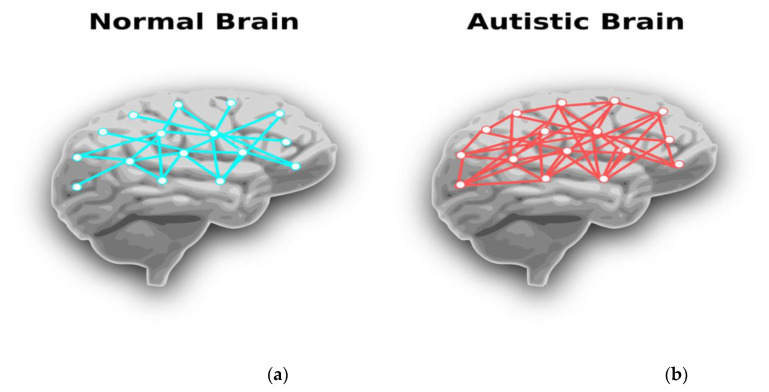
(**a**) Neurotypical brain and (**b**) ASD brain with denser neural connections.

Autism spectrum disorders (ASDs), with a reported prevalence in developed countries of around 2% [26], typically present within the first three years of life. ASDs are characterised by challenges in social interaction [27,28], speech and language delays, avoidance of eye contact, struggles to cope with changes in environment, the display of repetitive behaviours, and differences in learning profiles [26]. Children and adults with an ASD have a high frequency of anxiety and depression. Research into the pathophysiology of ASDs have revealed neurobiological differences between children with ASDs and those without (‘neurotypical’ children). Figure 3 highlights the excess neural connections between neurons in the brain of a child with an ASD compared to a neurotypical individual [29]. These excessive connections are thought to be secondary to reduced ‘pruning’ of damaged neuronal connections during brain development. This neuropathological difference is understood to result in disordered neural patterning across the brain and dysregulation in cognitive function coordination between different brain regions [30].

### 1.2. Personalised Assistive Tools Using Artificial Intelligence for Children with NDD

The prior summary highlights that NDDs, such as ASDs, ADHD and dyslexia, are globally prevalent conditions associated with poor learning outcomes and a high prevalence of comorbid MHDs. A recent study attests that more support strategies are required to help students with these conditions in their learning in mainstream schools [31]. There is a critical need to apply effective tools to improve learning outcomes. Personalised assistive educational tools might help improve educational outcomes, helping affected individuals to integrate better into society, reducing stigma, isolation and stressful events, such as bullying, that are known to be common triggers for suicide attempts [32]. Hence, Section 1 highlights the three main NDDs and the need for personalised assistive tools. Section 2 discusses instructional practices employed in schools, challenges encountered and the potential role of AI tools in addressing those challenges. Section 3 discusses the research methodology, while Section 4 provides a summary of AI tools developed and discusses the effectiveness of these tools for personalised education. In Section 5, the limitations of existing AI tools are discussed. In Section 6, the future AI tool that addresses the limitations of existing tools for personalised education is proposed. Chapter 7 concludes the review study.

## 2. Current Management Approaches for Children with NDD

### 2.1. Individualised Educational Approaches for a Child with ADHD

Inclusive approaches are recommended so that students with NDD can be included in mainstream schools wherever possible [33]. However, mainstream classroom settings can worsen symptoms in children with ADHD, especially when students are expected to sit still, remain quiet and stay focused [34,35]. Mainstream schooling without additional support can thus damage children’s self-esteem and negatively impact their relationships with teachers and peers [33]. Hence, to cater to the individual needs of these children, it is recommended that teachers employ individualised educational practices in the classroom [36,37]. Details of such individualised educational practices are listed in Table 1.

### 2.2. Individualised Educational Approaches for Children with Dyslexia

A multi-sensory approach, whereby information is shown simultaneously through varying channels, is reported to be preferable when teaching dyslexic children in schools [18]. Table 2 summarises strategies that have resulted in successful outcomes when employed in the school context for children with a diagnosis of dyslexia.

### 2.3. Teaching Support in Schools for Students with ASDs

Children with ASDs think, learn and behave differently from neurotypical children and differences in auditory processing, inspiration, emulation and organisation can hamper the learning success of children with ASD [38]. Hence, unique structured teaching strategies have been employed to cater to the different learning needs of ASD children, as listed in Table 3 [38].

### 2.4. Challenges in Implementing Individualised Learning Approaches in Schools

Despite evidence that individualised lessons are effective in helping children overcome their learning disabilities, teachers face several challenges in achieving this goal [40]. It takes a lot of time for teachers to provide differentiated instructions, ensure the child has understood them, and then achieve goals that have been set for individual students [40]. Many schools have shortages of appropriate learning resources. Therefore, an assistive learning tool that can be personalised for children’s individual learning challenges and needs would be very helpful for teachers and a great support in helping students meet their individual goals. Although learning a new tool and teaching children to learn the assistive tool may be time-consuming initially, the benefits of using such tools to support individualised learning and improve overall clinical and educational outcomes are clear.

### 2.5. Use of Artificial Intelligence in Therapies and Supportive Education of Children with Mental Disorders

#### 2.5.1. Conventional Methods Using AI

**Figure 4:**
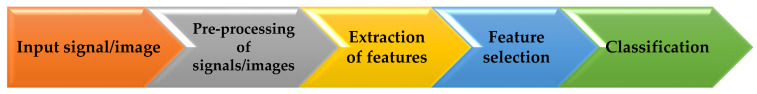
Sequence of steps for training a machine learning model.

Machine learning forms a part of artificial intelligence (AI) wherein the model is able to do tasks automatically, without needing any human interference. The conventional machine learning models are hence trained by the input data fed to them, after which these models are able to predict outcomes with high accuracies. Deep learning is a subfield of machine learning in which large data is used to train these models, which can also predict outcomes with high accuracies. Both models are commonly used in the diagnosis of some neurological disorders, such as autism [41,42], ADHD [43,44] and depression [45,46,47], with high accuracies. The models are either fed with images obtained from computerised tomography (CT), magnetic resonance imaging (MRI) and positron emission tomography (PET) scans or electroencephalogram (EEG) signals for the diagnosis of neurological disorders. Figure 4 shows the sequence of steps involved in training a machine learning model for diagnosis; after the input of signals or images, pre-processing takes place to clean the data, after which features are extracted and ranked thereafter, to obtain the most significant features, before they are finally classified into normal or abnormal classes. While the feature extraction and selection processes need to be done manually by the researcher for conventional models, these processes are done automatically in deep models.

#### 2.5.2. Advanced Methods Using AI

**Figure 5:**
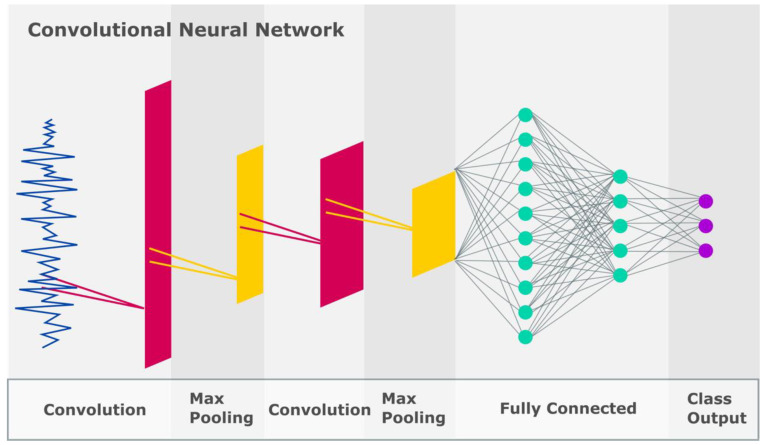
Illustration of the CNN model.

**Figure 6:**
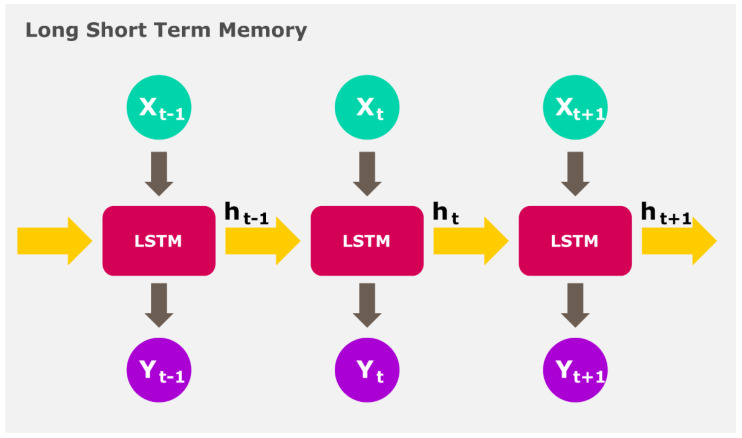
Illustration of the LSTM model.

**Figure 7:**
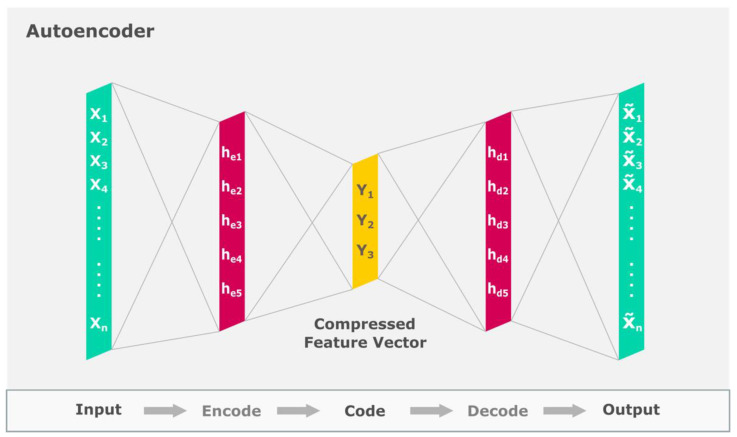
Illustration of the Autoencoder model.

Some frequently used deep models include the convolutional neural network (CNN) [48,49,50], long-short term memory model (LSTM) [51] and autoencoder [52], as depicted in Figure 5, Figure 6 and Figure 7, respectively. In CNN models, the input data is fed to the convolution layer. New feature maps are created in each successive layer wherein more robust features are extracted for forecasting. In the final fully connected layer, the data is classified [48]. The LSTM encompasses three main blocks of memory cells, input, forget and output gates, which are accountable for controlling the information stored, read and written on the cell, respectively, as data comes in [51]. The LSTM principally performs by remembering important information from preceding states and building upon them [51]. Encoders are arranged to form the deeper autoencoder model. Autoencoders perform by encoding unlabelled input data and rebuilding the data accurately thereafter. These models comprise the coding and decoding phases, wherein the same weights are used to encode the feature and rebuild the output [52].

#### 2.5.3. Importance of AI in Therapies and Supportive Education

AI has been used to assist in social skills training in children with ASDs to recognise and respond to social cues. Belpaeme et al. [53] used sensory features, such as facial expression, body movements and voice recordings, as inputs to a machine learning model (implemented in a robot) to analyse autistic children’s behaviour and engagement levels for therapy. These input features were then combined with target outputs, engagement markers in this case, to train the model. The study proved the potential for the robot to adapt to its interactant, hence influencing engagement in participants. In another study, Sanghvi et al. [54] used postural expressions, such as silhouette images of the upper body, as they played chess with a robot to analyse the engagement levels of autistic children. Another positive outcome was established through this study; the potential of integrating representative data (as described above) with an affect recognition model to act as a game mate for autistic children in the real world. In a different study, Kim et al. [55] used audio recordings as input features to analyse the emotional states of autistic children. These features were fed to the support vector machine model, integrated with a robot, to assess their social engagement as they played with the robots. This enhanced audio-based emotion forecast approach discusses the possibility of sustaining a more natural interaction between autistic children and the robot, hence allowing the robot to assess the engagement level of the children more accurately and modify its responses to maintain an interactive learning environment. Other researchers have explored different input features, such as facial expressions [56], body movements [57], bio signals [58] and vocalisations [39]. In a more recent study, Esteban et al. [59] explored input features, such as facial expressions, direction of look, body posture and voice tones, to a model within the NAO robot to assess the social engagement of autistic children. This study attests to the capability of robots to possess increased autonomy, so as to lighten the load of therapists.

Moving towards individualisation, Rudovic et al. [60] developed a personalised deep model, using coordinated video recordings of head and body movements, facial expressions and gestures, audio recordings and bio signals such as heart rate, electrodermal activity and body temperature, to assess the engagement of autistic children. The results reported that the model matched human experts, the prediction of affect and engagement with that of human experts, with an accuracy of about 60%, outperforming non-personalised machine learning solutions. In another study, a hybrid physical education teaching tool was developed, wherein speech recognition combined with artificial intelligence was used to construct a personalised voice interactive educational robot. The results showed that the robot was able to answer students’ questions, achieving a recognition accuracy of more than 90% [60]. Hence, the aforementioned studies affirm that AI is a promising avenue to improve social interaction and supportive education in children with mental disorders.

## 3. Materials and Methods

**Figure 8:**
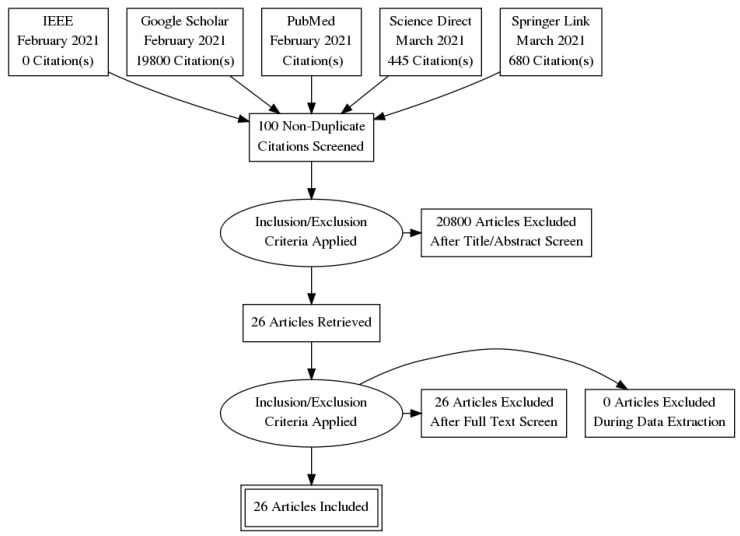
Flowchart detailing the use of PRISMA guidelines for selection of relevant articles.

The systematic review was conducted based on the Preferred Reporting Items for Systematic Reviews and Meta Analyses (PRISMA) guidelines to analyse the most relevant studies on assistive tools developed to address learning disabilities using machine learning models. The search was conducted between the years 2011 and 2021. The relevant journal articles were searched through the IEEE, Google Scholar, PubMed, Science Direct and Springer Link scientific repositories, as seen in Table 4. The Boolean search strings such as “machine learning,” “artificial intelligence tools,” “Autism spectrum disorder,” “Attention deficit hyperactivity disorder,” “dyslexia,” “students” and “learning” were used in various combinations. The relevant articles were gleaned from the various databases for this review, based on three primary processes from the PRISMA guidelines. First, a total of 20,926 articles were *identified* based on the Boolean search strings for autism spectrum disorder, attention deficit hyperactivity disorder and dyslexia. From the IEEE, Google scholar, PubMed, Science Direct and Springer Link repositories, 1, 19, 800, 0, 445 and 680 articles were retrieved, respectively, wherein most articles described the diagnostics/prediction/screening of ASD, ADHD, dyslexia or other neurodevelopmental disorders. Subsequently, articles were *screened* wherein duplicate and irrelevant articles were excluded based on the inclusion and exclusion criteria. The process culminated with the *selection* of the most relevant articles, which was set to 26. The flowchart detailing the processes involved in the retrieval of appropriate articles using PRISMA guidelines is shown in Figure 8.

The search was conducted between February to March in 2021. Studies were included if they met the following criteria:

They described the use of AI tools to help students with ADHD, Dyslexia and/or ASD in their learning:
They were published between the years 2011 and 2021.
They were published in a peer-reviewed journal.
They were published in English.

Studies were excluded if:

They described the use of AI tools to help students with other disorders apart from the NDDs ADHD, dyslexia and/or ASD.
The article was not published in English.
The article was not published in a peer-reviewed journal.
The article was published before 2011.

## 4. Results

### 4.1. Summary of Articles Collated

Table 5 presents the summary of articles that involve the use of AI tools (machine learning techniques) for teaching children with learning disabilities. Abstracts were first searched and then a full text review was conducted to ensure that the studies met the inclusion and exclusion criteria and that data could be extracted on the AI tool used, the features/model used for training, the type of technology and the learning area addressed (Table 5). Although not commonly reported, the effectiveness of any AI tools on students’ learning was also recorded.

### 4.2. Effectiveness of AI Tools for Personalised Education

Most AI tools that were used for learning, as shown in Table 5, have reported positive outcomes. For example, the ‘Child activity sensing and training tool’ for ADHD [69], which was tested in authentic situations, has been identified as having the potential to aid ADHD students in gaining attention in school settings, as well as to track their physical and physiological activities in real time [69]. Additionally, this tool has the potential to aid an ADHD student who has lost focus in his/her work. Furthermore, the ‘Emotify’ game developed for autistic students has obtained an accuracy of 72% in emotion recognition [83], which is a testament to its successful application. Additionally, the application has enabled participants to experience more engagement and exhibit higher behavioural intentions towards it. A study that used ‘Facesay’ games to aid autistic students [86] reported improvements in emotion recognition, social interaction, facial recognition, emotion recognition and social interaction in low-functioning and high-functioning autistic students. The application has also been found to be very promising, cost-effective and efficient for teaching effect recognition and mentalising constructs to high-functioning ASD students. The development of software games to help students with challenging behaviour of ADHD students has resulted in positive impacts [85] in addressing their moods, wherein a reduction in challenging behaviours of participants was reported when the games were used as an intervention to improve behaviours. Apart from the applications, the use of robots [84,85,92,93,102,103] and alternative communication devices [78,86] have been proven to be effective in improving focus, as well as math and social skills and in the teaching of ASD students, respectively. Hence, the abovementioned findings confirm the effectiveness of AI tools for personalised education.

## 5. Discussion of Main Findings and Results of Study

**Figure 9:**
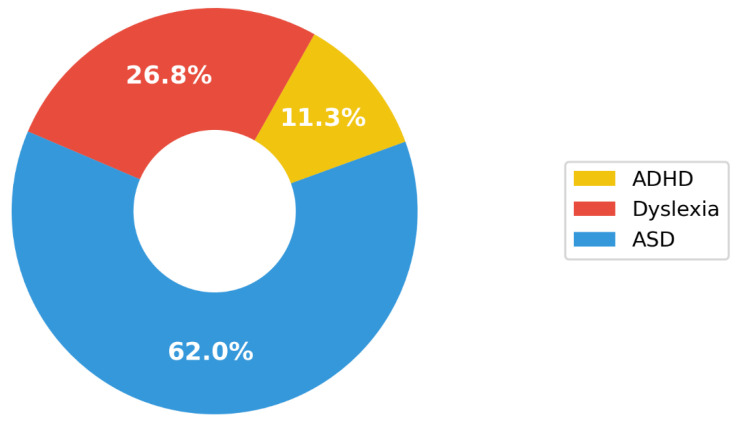
Pie chart representation of assistive tools used to aid in the learning of ADHD, dyslexia and ASD students.

**Figure 10:**
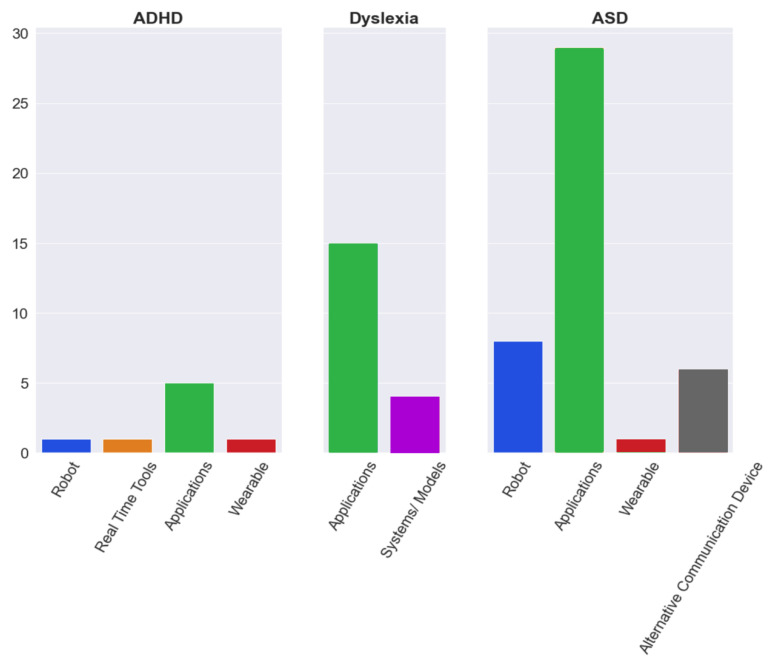
Bar graph representation of various assistive tools used to aid in the learning of ADHD, dyslexia and ASD students.

Figure 9, derived from Table 5 illustrates the distribution of assistive tools used to aid ADHD, dyslexia and ASD students in their learning. Figure 10, also derived from Figure 4, depicts the various assistive tools used to aid ADHD, dyslexia and ASD students in their learning. Hence, based on Figure 9 and Figure 10, it has been established through this study that most assistive tools have been developed to support ASD students in their learning and that the most prevalently developed tools to help ADHD, dyslexia and ASD students in their learning are application-based tools. Through this review, it has also been well-established that robots and application-based tools have been developed most to support ASD students, as compared to ADHD and dyslexic students, in their learning. This is in line with an interview conducted with educators in England on humanised robots discussing optimistic views shared by some educators [69]. These educators highlighted specific cases where robots were bound to be impactful, for example as “stepping stones” to social interactions and the capabilities of personalised robots to meet the individual learning needs of learners with ASDs. Furthermore, educators also felt assured that the predictability and constancy of behaviour in robots would benefit learners with ASD, facilitate their learning and diminish the burdens on them [69]. The results of our review also confirm that certain AI tools have shown positive outcomes and have been successful in certain educational settings. From our review study (Table 5), it is evident that mostly application-based tools and robots have been developed to aid students with an ASD in various aspects of their learning, from developing social and communication skills to daily living skills. Furthermore, it is noticeable that more types of technology have been used and hence more AI tools have been developed to aid students with ASD as compared to other learning disabilities (Figure 10). Perhaps this may be due to the challenges in learning, social communication and play skills that individuals with ASDs face. Additionally, from the results of our review (Table 5) and extant literature, it is evident that deep learning models are used to develop robots to learn a large variety of data due to the highly heterogenous nature of ASDs [105]. Similarly, application-based tools have also been used to address the learning needs of students with dyslexia. These tools possess special features that allow individualised learning for each student. A combination of wearable devices, robots and application-based tools have been developed to aid students with ADHD. However, from the results of our review (Table 5), it has been established that not all developed tools possess special features that allow personalisation. The learning needs of a student depend on the severity and precise nature of how that disorder affects them, and thus varies from one student to another. Hence, we deduce that along with the development of AI-based assistive tools, it is imperative that special features are entrenched that allow personalisation, such that the distinctive learning need(s) of every student is met [30]. Despite its advantages, using AI to develop such tools also displays some limitations, as discussed below.

### 5.1. Limitations of Existing AI Tools for Personalised Education

#### 5.1.1. Suitable Datasets

There is a scarcity of public databases because it is challenging to obtain data from children with certain NDDs because they frequently have difficulties in staying still. There is also limited data available that focus on the severity of such disorders. Many children have complex comorbidities, so developing personalised AI tools for such students can be challenging.

#### 5.1.2. Ethical Considerations

There are ethical concerns about using AI-driven applications in the development of personalised tools for learning. Educational assistive technologies are commonly used with students who are adolescents; hence, ethical concerns arise over privacy, data security and informed consent, which need to be mitigated [106]. For example, the information collected about a student should be reduced to include only information necessary for the intended purpose [106], i.e., to train the machine learning model. Data collection of the student should start only once the individual knows that data collection is taking place and has consented to the data collection [106]. Most importantly, educators should also ensure that students understand the consequences of using assistive technologies to protect their privacy and data [107]. To ensure the consent is valid, the teacher could describe the potential risks and benefits in a way that does not prompt a specific decision from those individuals who may be affected by the use of the technologies [107]. Additionally, it is imperative that educational technology companies comply with the relevant regulations; for example, the Children’s Online Privacy Protection Act [108] of the United States makes an attempt to determine the age of students who assess the company’s applications, programmes or extensions for student data collection. If the student is below 13 years old, parental consent is required for data collection [107].

Thus, it is important that privacy considerations are respected and that student data used to train machine learning models is secure according to local ethics board requirements [109]. Safeguards need to be in place to ensure data cannot be hacked [109], and educators should ensure ethical considerations are in place before any student data is collected, when the assistive tool is being used.

#### 5.1.3. Cost of Implementing AI Tools

Besides ethical issues, Tsai and Gasevic [110] corroborate other limitations of AI in the technical (system integration) [111] and financial aspects. The costs involved in installing, maintaining and repairing AI tools is another limitation of implementing these tools in classroom settings [112]. Hence, to make AI affordable and integrate it with ease, it is imperative for schools to assimilate AI technology in a cloud-based intranet [112]. This would be more cost-effective, as schools would just need to pay an affordable subscription cost monthly, wherein various aspects, such as the installation, storage space, specifications, maintenance and technical support would be taken care of [113].

#### 5.1.4. Information Loss

Another limitation is the possible loss of pertinent information when AI tools require repair [102]. Information such as students’ learning data may be compromised, hampering the training of AI models or assessment of existing tools. This could be averted by adopting a cloud-based approach to safeguard information [112].

### 5.2. Cloud Computing in Schools

In recent years, cloud computing is progressively becoming popular in delivering technology in the education domain [114]. Cloud computing also allows schools to use less potent computers to assess the cloud, hence reducing computing costs [114]. However, cloud computing has potential disadvantages, and privacy, security and legal (policies) issues need to be addressed to successfully implement a cloud-based system [114].

#### 5.2.1. Advantages of Using AI in Cloud Computing

The processing of information and resources, comprising the storage, sharing, backup and recovery of substantial information and resources, is a crucial aspect of AI-based technology [115]. Hence, integrating AI technology in cloud computing is beneficial as the internet and central remote servers are used to sustain information, resources and applications, resulting in effective computing as storage, processing, memory and bandwidth are centralised [115]. Similarly, backup of information can be done effortlessly by cloud-based applications, unlike traditional methods of using hard discs that are time-consuming, expensive, contain limited capacity and can require laborious maintenance [116].

**Figure 11:**
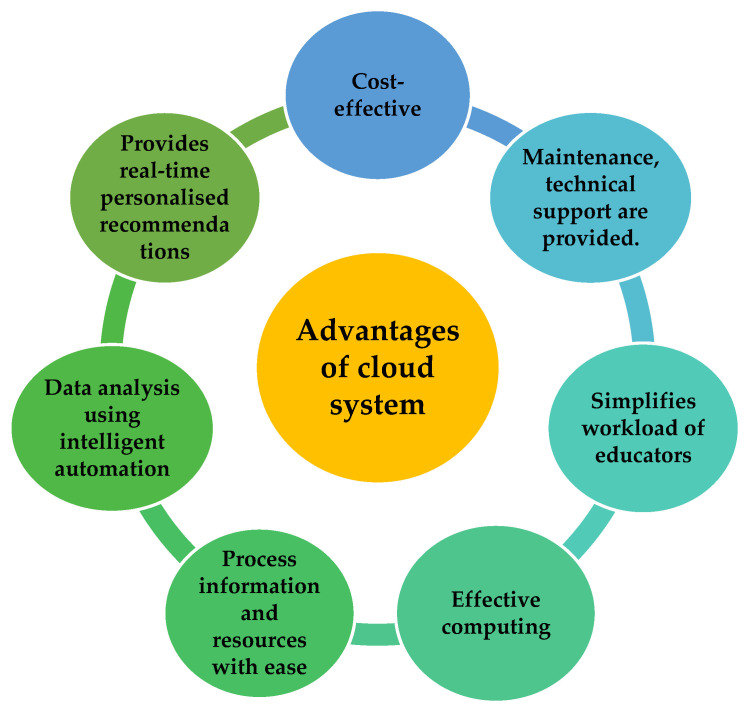
Benefits of using the cloud system in schools for personalised education.

Merging the AI tool in the cloud is beneficial as it is cost-saving, wherein costs related to hardware and maintenance or on-site data centres are eliminated [117]. The AI tool is also able to computerise intricate and repetitive tasks to enhance productivity and perform data analysis using intelligent automation; hence, it does not require any human intervention. The AI-based tool is also able to perform data analysis rapidly and provide deeper insights and hence is competent in offering real-time personalised recommendations to users [117]. AI tools are able to simplify how data is used, modified and handled and thus improve data management when imbedded in cloud systems. AI-powered network security tools need to be used to improve security to protect critical data when imbedded in cloud systems. Using the cloud server is also beneficial as it is part of the internet of things (IoT), where the data on shared networks [118] enables faster and more accurate outcomes. It also optimises equipment and usage of resources [118] that aid in managing situations better, such as being able to develop personalised tools for individuals. The core advantages of using cloud computing in school settings for personalised education are summarised in Figure 11, as seen below.

#### 5.2.2. Disadvantages of Using AI in Cloud Computing

While AI in cloud computing may generally lower costs, a well-trained staff needs to be employed to operate the complex AI system and this may incur some cost. The cloud system would also require continuous internet connection to work well; weak internet access can impair the benefits of the cloud system [117]. Additionally, since data processing is speedier in the cloud than without using the cloud (methods discussed in Table 5), a time lag between transferring data to the cloud and receiving responses may be present. Privacy policies need to be complied with for data security when AI is used in cloud computing [117].

Based on the discussion above, it is apparent that there are downfalls to using AI in cloud computing. For instance, as mentioned earlier, data safety and security are the major concerns of using AI tools/AI in cloud computing. To tackle cyber security issues, decentralisation should be used wherein information acquired is split into parts and stored in various parts of the network instead of storing the entire information in a central server [119]. Additionally, user privacy methods, such as including a physical layer that conceals certain measurements of users [120] or enhanced security keys that create provisional identifications [121], should be employed within the cloud system to tackle privacy issues. Ethical considerations should also be followed. Despite the limitations, it can be observed that the benefits of integrating the proposed AI tool in the cloud outweigh the limitations, especially in the development of personalised tools.

## 6. Proposal for a Future AI Tool

**Figure 12:**
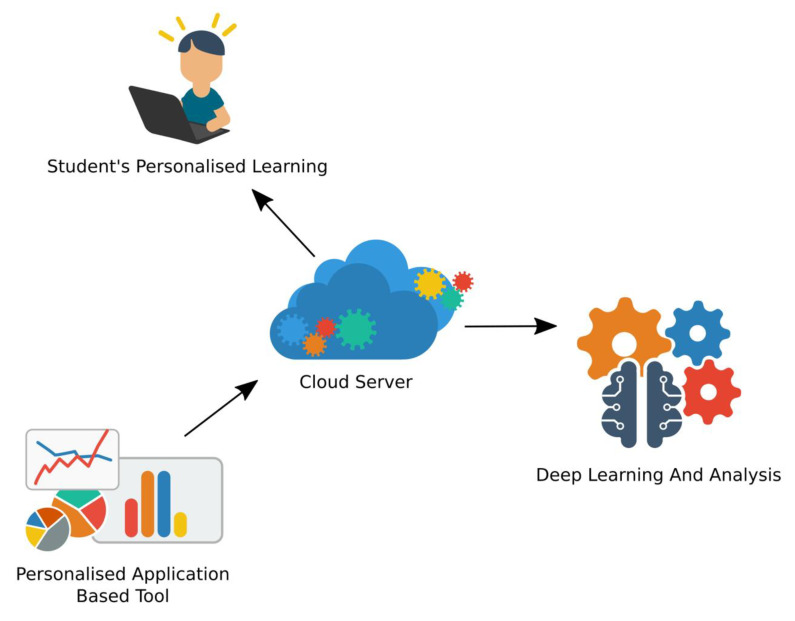
Proposed AI-based tool for personalised learning.

Presently, machine learning techniques have not been explored in cloud-based applications for personalised learning tools. Hence, in our future work, we will be using deep learning techniques to develop a unique cloud-based model or an application-based tool that enables personalisation and serves as an assistive tool for teachers or other adults who assist in learning. Sizeable data comprising input features, such as facial expression images, speech signals, bio signals and clinical information, such as age, gender, genetic history, and so on would be used to develop the personalised model. The data obtained from the individual user will be sent to the trained deep learning model, which will be kept in the cloud server. The model will then be able to predict the user’s learning needs accordingly and provide personalised learning to suit the child’s learning needs. The proposed tool will also have decentralisation and user privacy methods implemented in it. The proposed AI-based tool for personalised learning is shown in Figure 12.

## 7. Conclusions

This review highlights that despite being in its infancy stage, assisted tools have been proposed to address the learning needs and quality of life of children with the most common NDDs: ADHD, dyslexia and ASDs [122]. The majority of the existing works using AI have focused on ASD. It is clear that more work needs to be done on the development and evaluation of assistive technologies for children with a range of NDDs [62]. The studies done to date have shown that AI-assisted tools have shown positive impacts on student’s learning, and have been found to be acceptable by teachers, parents, special educators and therapists and feasible to implement in their teaching or therapeutic practices [122]. AI techniques have been reported to assimilate the independence of user’s actions and enable children with learning difficulties to achieve their individual learning goals [122]. However, as discussed previously, our review reports that the existing AI assistive tools exhibit some limitations, so more work still needs to be done to ‘mainstream’ such approaches and maximise their impact. For example, the AI tools reported here are not embedded in cloud systems, hence limiting their ability to provide real-time suggestions for personalised learning. AI-based tools in the cloud system, such as digital applications, could be a major advance facilitating the provision of personalised specialist education and learning for affected individuals in real time.

## Figures and Tables

**Table 1:** Individualised educational practices in schools for children with ADHD.

Learning Area	Intervention
Reading comprehension	Establish a sustained silent reading time daily. Allow the child to read a book silently while listening to the teacher reading the story to the whole class. Getting the child to make a storyboard, retell a story during story sessions, role-play characters in a favourite story. Allowing the child to play board games/computer games to enhance reading comprehension skills. Maintaining a word-bank book for words that are hard to read. Providing students with another set of books to be read at home [36]
Phonics	Teaching children simple reminders on how to learn tougher phonics. Teaching children how to recognise word families for phonetic concepts. Allowing students to play board games, such as Bingo or computer games, to enhance phonics. Using picture–letter charts for children who can identify sounds but not letters [36]
Writing	Using storyboards to teach students to recognise parts of a story for writing. Creating a post-office in the classroom for students to write and receive letters from their teachers and peers. Using tape recorders to dictate as an alternative to writing or having teacher/peer to write for students who would tell the story [36]
Spelling	Aligning spelling words to frequently used words by children everyday. Partnering the child with another peer to encourage each other to quiz on spelling words. Using colour-coded letters to help students spell difficult words. Combining movement activities with spelling lessons [36]
Handwriting	Using special writing paper or teaching how to use a finger spacing to space out each word when writing. Teaching handwriting skills through structured programmes [36]
Mathematics computation	Using mnemonics to describe fundamental steps easily for Maths computation. Colour-coding arithmetic symbols to provide visual cues, allowing students to use calculators for basic computation, using computer/board games for practicing computations [36]

**Table 2:** Individualised educational practices in schools for children with dyslexia.

Learning Area	Intervention
1. Failure in reading, grouping letters in words.	Using visual perceptions, such as signage or touching letters, to help in reading. Providing simultaneous stimulation of each ear using different sounds [23]
2. Phonology	Employing strategies that help phonological processing, such as ‘minimal pairs,’ ‘common syllable words,’ and ‘vocal syllabification’ [23]
3. Grammar	Using grammatical processing strategies, such as ‘syllabification,’ ‘declension of nouns,’ ‘stress,’ and ‘nouns’ [23]
4. Writing	Using the syntactic approach to teach punctuation and sentences/paragraphs. Using the ‘segmentation with highlighting’ technique for sentence and text segmentation [23]

**Table 3:** Individualised instructional practices in schools for children with ASD.

Structured Teaching Strategies	Intervention
Physical structure	Establish a supportive classroom environment by creating clear physical or visual boundaries such that expected behaviours for each defined space can be taught and reinforced [39]
Reducing auditory and visual disturbances	Too much auditory or visual stimuli may hamper processing power; hence, unnecessary distractions are removed in classrooms to help students focus better on concepts taught [39]
Visual schedules	Implementing visual schedules for the day (instead of using verbal probes), according to the learning needs of each student to enhance student independence and engagement during lessons [39]
Work system	Implementing a work system for any type of educational activity helps to organise the student by providing a systematic work routine [39]
Visual structures	Adding a physical or visual aspect to some tasks to help students understand better how an activity needs to be completed [39]

**Table 4:** Results of the Boolean search string for the respective repositories.

Boolean Search String			
Database	Title	AND [Title/Abstract/Full Text]	No. of Articles
IEEE	“Autism spectrum disorder”, AND/OR “Attention deficit hyperactivity disorder” AND/OR “dyslexia”, Artificial intelligence AND/OR tools, students AND/OR learning	Machine learning, Neural networks, deep learning	Autism: 0, ADHD: 1, Dyslexia: 0
Google Scholar			Autism: 12 100, ADHD: 3900, Dyslexia: 3800
PubMed			Autism: 0, ADHD: 0, Dyslexia: 0
Science Direct			Autism: 176, ADHD: 172, Dyslexia: 97
Springer Link			Autism: 357, ADHD: 179, Dyslexia: 144

**Table 5:** Review of AI assistive tools used to address learning disabilities of students with NDDs.

Author/Year	AI Tool	Features/Model Used for Training	Type of Technology	Learning Area Addressed	Effectiveness
**AI Assistive Tools Used to Teach Students with ADHD.**					
2014 [61]	KAR robot	-	Assistive technology	Improve social skills via storytelling.	Improves children’s cognitive performance.
2015 [62]	Child activity sensing and training tool	- 42 features (users’ physical and physiological) - Machine learning algorithm (not specified)	Real time assistive technology	Real-time assistive tool that tracks activities and helps students sustain attention.	An assistive intervention that is based on a smartphone and has the potential to aid a child with ADHD who has lost focus in his/her work.
2018 [63,64]	WatchMinder vibrating watch	- Sensors that collect activity and behaviour data - Bespoke algorithm/application	Wearable technology	Helps to send constant reminders to students to refocus on their work.	The watch has been effective as a simple memory aid for ADHD children with the auditory or vibrating alarm feature. The watch has been found to be affordable, durable, dependable and effective by users [65]
2018 [63]	Speech recognition software (Dragon Naturallyspeaking(Dragon Sytems company, United States, version 15, /Voice Finger/ViaTalk (LLC Company, New York/Tazti (Voice Tech Group company, United States)	- Audio data - Deep learning models (spectrograms/filter banks [66]	Assistive technology	Replaces writing activity with speech to allow students to express themselves efficiently without tiring themselves	Dragon Naturallyspeaking, Voice Finger, Via Talk, Tazti softwares have been reported to be beneficial to students with ADHD and resulted in improvement in the areas of writing, reading and spelling [65]
2018 [63,67]	Talking calculators	- User’s data such as pressing of numbers - Built-in speech synthesiser	Assistive technology	Helps students hear and process numbers easily for mathematics.	Students are able to complete assessments faster with the help of the calculator and has helped students gain independence [67]
**AI assistive tools used to teach students with Dyslexia.**					
2013 [68]	Intelligent dyslexic system	Machine learning algorithm, visualisation concept	Assistive technology	Helps students gain knowledge on alphabets and letters	The technology has the potential to improve the reading and writing skills of students.
2014 [69]	Agent DYSL adaptive reading system	Machine learning algorithm, Mel-frequency cepstral coefficients, discrete cosine transform	Assistive technology	Enables the personalisation of reading environment of Greek students.	Students’ reading pace and accuracy were increased.
2015 [70]	Computer-based learning model	Machine learning technique	-	Explores the use of machine learning method to improve effectiveness of learning process.	-
2017 [71]	Applications for reading and writing (Learning Ally, Natural reader, dyslexia quest, sound literacy, ginger page, v books pdf voice reader, openWeb, reading intro by OZ phonics, OCR instantly pro, MindMeister)	Generation of audio files, pytorch deep convolutional text-to-speech models (PytorchDcTts)	Digital application	Helps students with reading and writing skills.	Audiobooks, such as learning Ally, has enabled students to gain confidence, independence and success [72].
2018 [73]	DIMMAND, capturaTalk application	- Chatbots	Digital application	Provides tailored interventions for difficulties encountered in literacy.	Information is not available.
2020 [74]	Voice dream reader, natural reader, web reader	- Text, audio data - Voicebot	Digital application	Helps with building reading skills.	E-readers have been found to generally improve reading speed and comprehension as compared to reading on paper [75].
2020 [76,77]	DytectiveU	- Learning patterns of students - Support vector machine algorithm	Digital application	Provides personalised game-based exercises to enhance specific cognitive skills related linked to dyslexia.	The DytectiveU application is reported to be able to offer students a variety of actions that are helpful in the learning of reading and writing [78].
2020 [79]	Generative adversarial network	Conversion of image/speech to text	Assistive technology	Converts natural language text to images to aid students in their learning.	-
**AI assistive tools used to teach students with ASD.**					
2011 [80]	LIFEisGAME game	- Shape and appearance-based facial features - Second feedback loop, visual input from player obtained through webcam	Digital application	To teach students to recognise facial emotions.	Information is not available.
2017 [81,82]	‘Empower me’ application	- Emotion recognition features - Google smartglass, augmented reality environment, web-based dashboard to monitor progress	Wearable technology	Encourages social interaction between user and peers/educators.	Students were able to improve their social skills using the Google glass. It was also reported to be fun, useful and engaging [83].
2018 [84,85]	Kaspar robot	- Sensory data - Reinforcement learning algorithm [86]	Assistive technology	Helps enhance social interaction skills.	The human-like body and features of Kaspar have been reported to help an ASD student to be more interactive [87].
2018 [88]	ABA flashcards- Emotions, Autism emotion, conversation builder, emotions and feelings- autism, Find me, Kid in storybook maker, learning with Rufus, Look in my eyes: Train engineer, Model me going places 2, Pictello, Social stories, Special stories, The social express, Toca Boca	- Deep learning/machine learning algorithms using unstructured data	Digital application	Teaches social skills	Information is not available.
2018 [88]	ABA find it, Agnitus, Autism learning games- camp discovery, Intro to letters, Intro to Math, Math Bingo, Pop Math, Starfall ABC, Word wagon	- Deep learning/machine learning algorithms using unstructured data	Digital application	Helps in different learning areas	The camp discovery enabled participants to show high learning rates over a short period of time. It has been suggested that the application teaches the selected skills effectively [89].
2019 [90]	Emotify game	- Audio features, such as pitch of voice - Speech data to train Random forest classifier	Digital application	Helps students to recognise and express feelings.	The application caused participants to experience more engagement and exhibit higher behavioural intentions towards it [91].
2019 [92,93]	Milo, NAO, Pepper, Aisoy 1, Keepon robots	- Supervised machine learning algorithms; generalised models trained on users’ data and individualised models trained on initial subclass of users’ data [94]	Assistive technology	Helps build social and communication skills.	Social robots, such as NAO, have been reported to improve social skills in students, especially in terms of eye contact and concentration. Nonverbal children also reportedly started pronouncing some words [95].
2020 [88,96]	GoTalks speech generating device, AAC speech buddy, Proloquo2go, talking Larry, Touch chat HD, VAST autism 1-Core	- Behavioural features, such as grabbing, vocalisations - Deep learning/machine learning algorithms, neural networks [97]	Augmentative/alternative communication device	Helps with building communication skills.	Review studies report that high-technology speech generating devices are very effective in teaching manding, intraverbal and multistep tacting to ASD students [98].
2020 [96,99,100]	Facesay games	- Scores on social interactions - Facial expression recognition techniques, interactive environment with lifelike avatars	Digital application	Software games help recognise behavioural and emotional clues and enhance social skills.	Facesay application is found to be very promising, cost-effective and efficient for teaching affect recognition and mentalising constructs to high-functioning ASD students [101].
2020 [102]	Personalised ‘Kiwi’ robot for learning	- Video and audio data, such as eye contact and verbal dialogue - Supervised machine learning algorithms; generalised models trained on users’ data and individualised models trained on initial subclass of users’ data [94]	Assistive technology	Adapts lessons according to students’ changing needs.	Kiwi robot has been reported to improve the Maths skills and social skills in ASD students who were part of the study group [103].
2020 [96]	Life skills winner application	- Deep learning/machine learning algorithms using unstructured data	Digital application	Teaches students daily living skills through the application.	Information is not available.
2020 [103]	PvBOT robot	LEGO Mindstorms EV3 model	Assistive technology	Helps to teach students ‘place value’ concept in Mathematics.	PvBOT is helpful in motivating students to pay attention and stay focused for a longer period.
2021 [104]	Squizzy educational software	Scrum methodology	Assistive technology	Helps children stay focused during activities that involve cognition, such as colour selection or using pictures.	Effective in the cognitive aspect of therapy.
